# *Announcement*: Sexually Transmitted Diseases Awareness Month — April 2017

**DOI:** 10.15585/mmwr.mm6613a4

**Published:** 2017-04-07

April is Sexually Transmitted Diseases (STDs) Awareness Month. This year, CDC is dedicating the month’s social media and web-based communication outreach activities to syphilis prevention. This observance provides an opportunity to share prevention messages and important resources with health care providers and affected populations.

U.S. surveillance data for 2015 indicate a sharp increase in reported syphilis cases: 23,872 primary and secondary syphilis cases were reported, for a rate of 7.5 cases per 100,000 persons, representing the highest annual number and the highest rate of reported syphilis cases in approximately 20 years and a 19% increase since 2014 (*1*). Primary and secondary syphilis are the earliest stages of infection, are indicators of incident infection, and differ in signs and symptoms. Primary stage signs/symptoms include a painless sore or sores at the site of infection. Secondary stage signs/symptoms can include skin rash, swollen lymph nodes, and fever.

From 2014 to 2015, syphilis rates increased in every region, a majority of age groups, and almost every racial/ethnic group. Syphilis rates are particularly high among gay, bisexual, and other men who have sex with men (MSM), who accounted for 82% of cases where the sex of sex partner is known. Approximately half of MSM with syphilis are also living with human immunodeficiency virus (*1*). Syphilis rates also increased 27% among women from 2014 to 2015, which has resulted in a surge in the number and rate of infants born with congenital syphilis. In 2015, the number of congenital syphilis cases was the highest it has been since 2001 (*1*).

Information and resources for persons, health care providers, and prevention partners is available at https://www.cdc.gov/std/sam/.
